# Hide or die when the winds bring wings: predator avoidance by activity shift in a mountain snake

**DOI:** 10.1186/s12983-023-00497-w

**Published:** 2023-05-16

**Authors:** Dávid Radovics, Márton Szabolcs, Szabolcs Lengyel, Edvárd Mizsei

**Affiliations:** 1 Conservation Ecology Research Group, Department of Tisza Research, Institute of Aquatic Ecology, Centre for Ecological Research, Bem Tér 18/C, Debrecen, 4026 Hungary; 2 Department of Ecology, University of Debrecen, Debrecen, Hungary; 3 Kiskunság National Park Directorate, Kecskemet, Hungary

**Keywords:** Prey capture, Thermoregulation, Basking, Serpentes, Thermal niche

## Abstract

**Background:**

Understanding predator–prey relationships is fundamental in many areas of ecology and conservation. In reptiles, basking time often increases the risk of predation and one way to minimise this risk is to reduce activity time and to stay within a refuge. However, this implies costs of lost opportunities for foraging, reproduction, and thermoregulation. We aimed to determine the main potential and observed predators of *Vipera graeca*, to infer predation pressure by estimating the incidence and the body length and sex distribution of predation events based on body injuries, and to assess whether and how the activity of *V. graeca* individuals is modified by predation pressure.

**Results:**

We observed n = 12 raptor bird species foraging at the study sites, of which *Circaetus gallicus*, *Falco tinnunculus* and *Corvus cornix* were directly observed as predators of *V. graeca*. We found injuries and wounds on 12.5% of the studied individuals (n = 319). The occurrence of injuries was significantly positively influenced by the body length of vipers, and was more frequent on females than on males, while the interaction of length and sex showed a significant negative effect. The temporal overlap between predator and viper activity was much greater for the vipers’ potential activity than their realised activity. Vipers showed a temporal shift in their bimodal daily activity pattern as they were active earlier in the morning and later in the afternoon than could be expected based on the thermal conditions.

**Conclusion:**

The time spent being active on the surface has costs to snakes: predation-related injuries increased in frequency with length, were more frequent in females than in males and occurred in shorter length for males than for females. Our results suggest that vipers do not fully exploit the thermally optimal time window available to them, likely because they shift their activity to periods with fewer avian predators.

## Background

Understanding predator–prey relationships is fundamental in many areas of ecology and conservation. Knowledge of these interactions is essential for mapping trophic networks, understanding community organisation and structure, demography, behavioural strategies, evolutionary processes, for threatened species conservation and conservation planning [1–3]. Predation has strong direct and indirect effects on prey populations [4–7], and predation pressure as a selective force has triggered physiological, morphological and behavioural adaptations in prey species [8, 9] such as camouflage colouration and morphology, bad taste or poisons, signalling or mimicking dangerous model species and many other active behaviours facilitating predator avoidance, e.g. escaping or counter-attacking [10]. Predator-avoidance behaviours have unambiguous short-term benefits in terms of survival but the long-term effects on fitness components vary. The activity pattern of prey is often influenced by predator activity, which can lead to a trade off with other actions in prey species [11]. Reduced activity is associated with increased survival in prey species, but it also decreases time spent on feeding, foraging success, growth rate and/or reproductive success. The reproductive status of individuals can also influence predation risk. For example, in viviparous snakes, mortality can be either lower or higher for pregnant females than for other individuals, which can lead to biased sex ratio within populations [12, 13]. Predation risk also shows temporal patterns over daily, lunar and seasonal cycles, depending on the environment and activity of both predator and prey [14]. Further, environmental changes, interruption of natural processes or arrival of a novel predator can lead to population decline of prey, and eventually to extinction in extreme cases [15].

The behavioural traits of ectotherms depend on body temperature [16], which in turn depends on the available environmental temperatures and behavioural choices related to thermoregulation. Ectotherms such as reptiles attempt to keep their body temperature in a narrow optimal range to optimise physiological processes [16–19]. The two main behavioural options to regulate body temperature and to keep body temperature within the thermal window bounded by thermal tolerance are the timing of activity [20] and the choice of microhabitats from the thermal landscape [21]. In temperate climates, the preferred body temperature can mainly be reached by basking, with associated costs of time and energy required for thermoregulation and of risks of predation [22, 23]. Because basking time increases the chance of being noticed by visually searching predators [23], as an anti-predator strategy, activity time can be reduced by staying within a refuge to minimise the risk of predation, however this implies costs of lost opportunities for foraging, reproduction, and thermoregulation [7, 24]. Avian predators usually have higher success in hunting snakes than mammals have, possibly due to better detectability by visual searching than by olfactory searching and to the poorer escape ability of snakes from predators searching for them from a distance [25].

Reptiles are among the most threatened vertebrates and are known to decline globally and in Europe [26, 27]. The meadow and steppe vipers (*Vipera ursinii* complex) are among the most threatened reptiles: lowland populations of this complex have lost almost all of their habitats due to the transformation of grasslands to croplands and have become extinct in a large proportion of their former distribution, while alpine populations are threatened by overgrazing and climate change [28]. In addition, meadow viper populations with low densities are also threatened by high predation pressure [29].

The general aim of this study was to assess predator–prey relationships involving visually foraging bird species as predators and the Greek Meadow Viper *Vipera graeca* [30], a rare, globally endangered, cold-adapted, mountain-dwelling venomous snake as prey. We specifically aimed to determine the main potential and observed predators of *V. graeca*, to estimate the incidence and the body length and sex distribution of predation events based on body injuries to infer predation pressure, and to assess whether and how the activity of *V. graeca* individuals is modified by predation pressure. To these ends, we surveyed predators in 14 of 17 known populations of *V. graeca*, examined *V. graeca* individuals for signs of predation-related injuries, and collected observational data on the daily activity of predators in the two largest *V. graeca* populations. We used thermobiological measurements (*V. graeca* preferred body temperature, environmental temperature) to estimate the thermal niche and potential activity window of *V. graeca* and compared the potential and observed activity of *V. graeca* and both against predator activity to study predator avoidance.

## Results

**Fig. 1:**
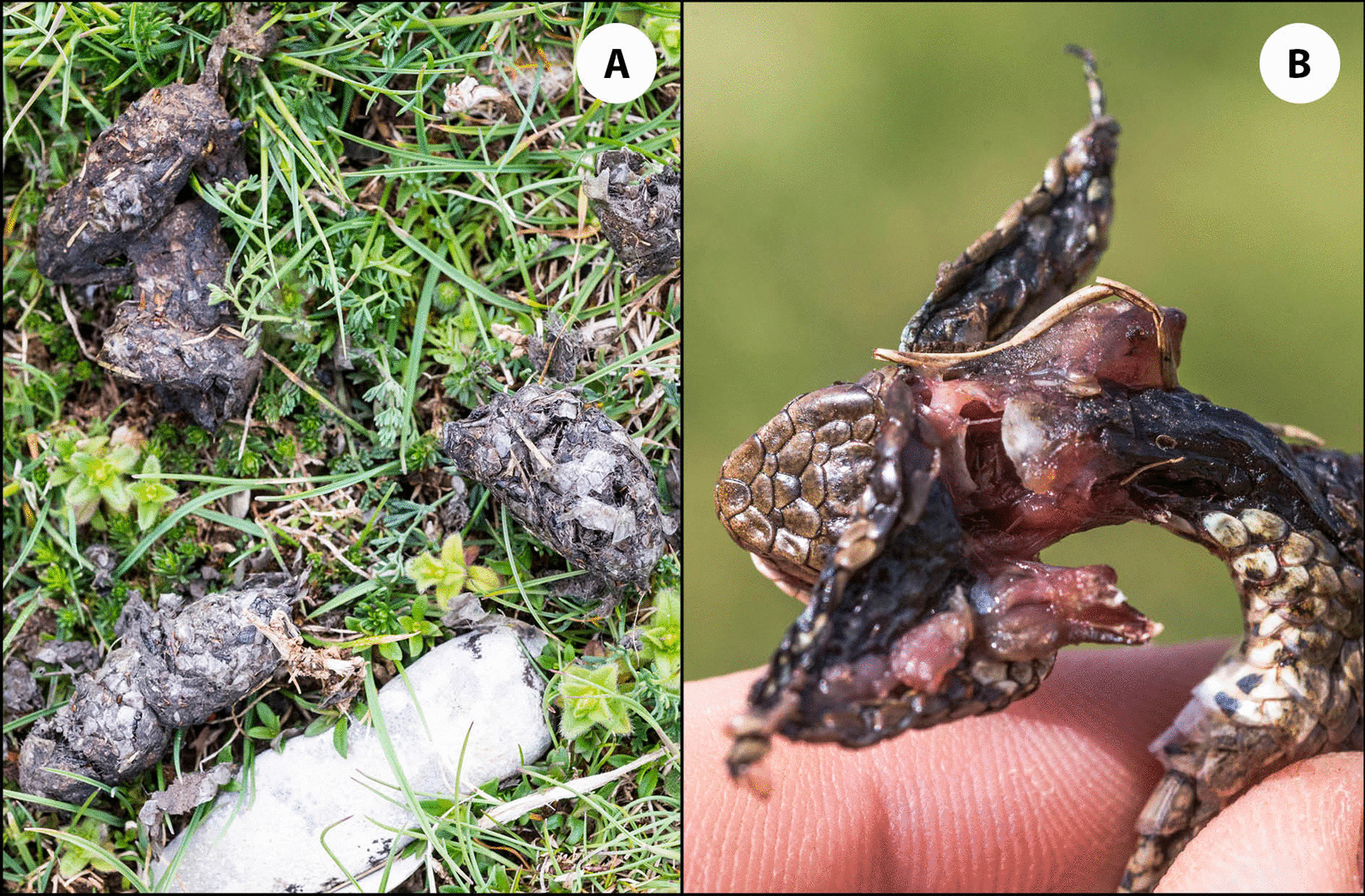
Pellets produced by *Circaetus gallicus* containing remains of *Vipera graeca*. Freshly killed *V. graeca* found by accidentally disturbing a feeding *Falco tinnunculus*

We observed 12 raptor bird species in the viper habitats, of which *Falco tinnunculus*, *Circaetus gallicus* and *Buteo buteo* were the most common. We collected evidence on predation on *V. graeca* by *C. gallicus* (pellets, n = 5), *F. tinnunculus* (pellets and direct observations of predation event, n = 4) and *Corvus cornix* (direct observation, n = 1) (Fig. 1). Based on a review of the literature, reptiles make up more than 10% of the diet of five raptor species that were regularly observed in the study areas (*Aquila chrysaetos*, *B. buteo*, *C. gallicus*, *F. tinnunculus*, *Hieraaetus pennatus*; Table 1).

**Table 1:** Species of birds of prey observed in the habitats of *Vipera graeca* and portion of reptiles in their diet.

Raptor species	Presence at Greek Meadow Viper habitats														Reptile % of diet	Reference for diet
	Av	Dh	Gr	Ka	Ku	La	Ll	Lu	Ne	Sh	To	Tr	Ty	Va		
*Accipiter brevipes*		+							+		+				−	
*Accipiter gentilis*							+								0.1%	[31]
*Accipiter nisus*						+									0.0%	[32, 33]
*Aquila chrysaetos*		+	+		+		+		+		+	+	+		11.1%	[34–40]
*Hieraaetus pennatus*			+				+						+		12.0%	[41–43]
*Buteo buteo*	+				+	+			+		+	+	+	+	20.63%	[44–52]
**Circaetus gallicus**		+	+	+	+	+	+	+	+		+	+	+	+	97.5%	[53–58]
**Corvus cornix**		+			+		+				+	+		+	−	[59]
*Falco naumanni*			+				+								0.1%	[60]
*Falco peregrinus*							+						+		0.12%	[61]
*Falco subbuteo*				+											0.0%	[33]
**Falco tinnunculus**	+	+	+	+	+	+	+	+	+	+	+	+	+	+	22.02%	[44–52, 62]

Species preying on *V. graeca* are highlighted in bold letters

**Fig. 2:**
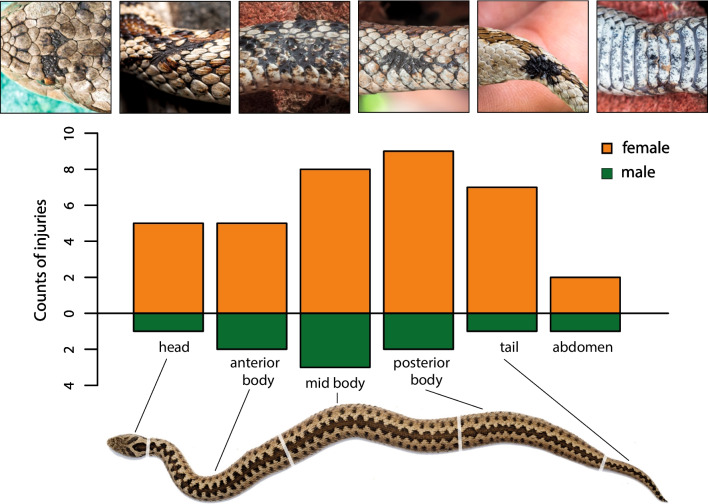
Frequency of predator-caused injuries on the different body parts of *Vipera graeca*. Photographs in the upper row are examples for these injuries

We examined 319 V*. graeca* individuals in 14 populations (mean ± S.E. 22.8 ± 5.27) for injuries. The apparent sex ratio was 0.64:1 males to females for all individuals and 0.51:1 for adults. Mean SVL of males was 244 ± 4.87 mm and 301 ± 5.11 mm of females. Females were significantly larger than males (W = 5341, *P* < 0.0001). We found injuries and wounds on 40 individuals (12.5%) The majority of these injuries were on the middle and the posterior halves of the body, including the tail (Fig. 2).

**Fig. 3:**
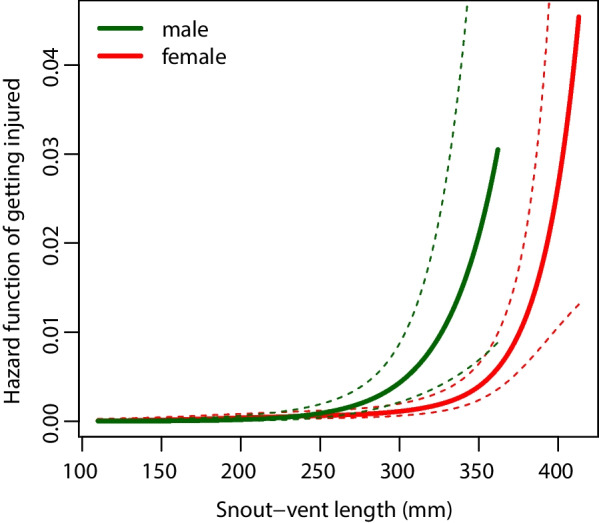
Hazard function for *Vipera graeca* (dashed lines indicate 95% confidence intervals)

The presence-absence of injuries/wounds on vipers’ body were significantly positively influenced by SVL (estimate = 6.920 ± 4.967 SE, Z = − 8.403, *P* < 0.0001) of viper individuals and were more frequent on females than on males (estimate = 19.431 ± 2.998 SE, Z = 6.481, *P* < 0.0001). The interaction of SVL and sex showed a significant negative effect (estimate = − 3.365 ± 0.533 SE, Z = − 6.318, *P* < 0.0001), as expected, because injuries were less frequent in males. Hazard functions showed that males obtained injuries at shorter SVL than females (Fig. 3).

**Fig. 4:**
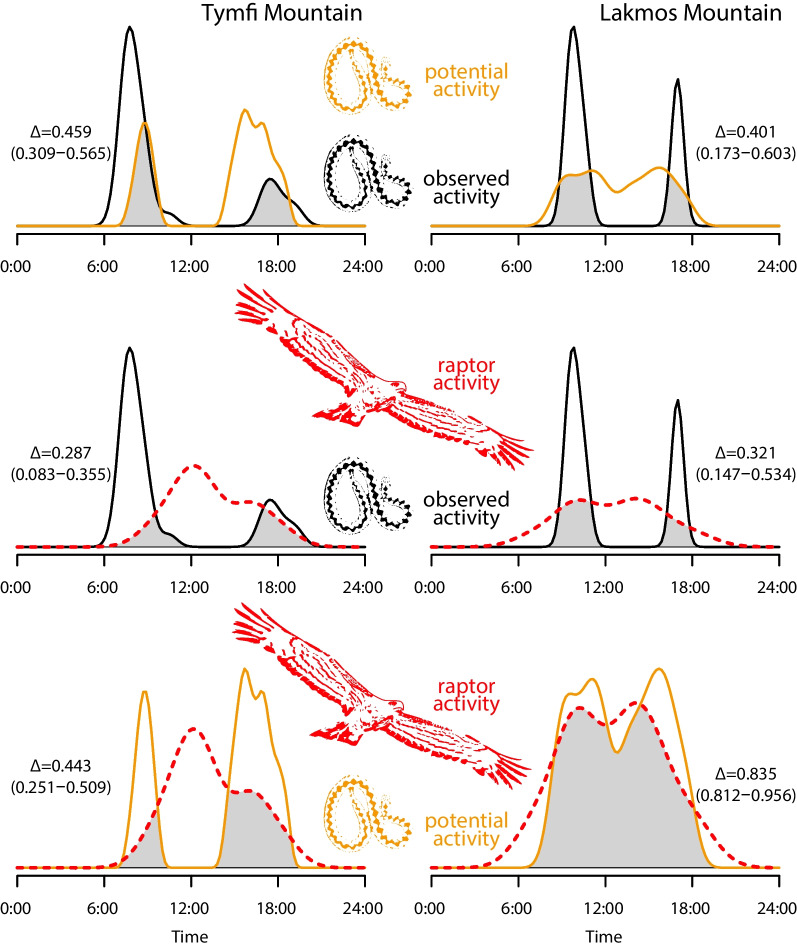
Observed and potential daily activity pattern of *Vipera graeca* and its’ predators. The coefficient of overlap (Δ) is accompanied by the 95% confidence limits in parentheses

Observation times of 38 individuals in two populations (Tymfi and Lakmos mountains) showed that the diurnal activity of *V. graeca* was bimodal, with one peak in early morning and another peak in late afternoon (Fig. 4). The comparison of the observed viper activity and the potential activity estimated based on environmental temperature and thermoregulation showed that the observed morning activity peak was earlier than could be expected based on the environmental temperature and preferred T_b_, and the observed afternoon activity peak was later than expected (Fig. 4). The observed and potential activity of vipers showed moderate overlap (Fig. 4, Δ_Tymfi_ = 0.459, Δ_Lakmos_ = 0.401).

During data collection on raptor diurnal activity, we observed six bird species, *F. tinnunculus* (53.1% of 98 observations), *C. gallicus* (22.4%), *B. buteo* (18.4%), *F. peregrinus* (3.1%), *A. chrysaetos* (2.0%), and *H. pennatus* (1.0%). The activity pattern of raptors (data pooled across species) was unimodal, with a peak at mid-day (Fig. 4). The overlap between the activity of raptors showed significantly smaller overlap (W_Tymfi_ = 57,662, P_Tymfi_ < 0.0001; W_Lakmos_ = 2, P_Lakmos_ < 0.0001) with observed viper activity (Δ_Tymfi_ = 0.287, Δ_Lakmos_ = 0.321) than with potential viper activity (Δ_Tymfi_ = 0.443, Δ_Lakmos_ = 0.835).

## Discussion

Our study of predator–prey relationships involving *V. graeca* and its avian predators provided four key results. First, we detected a large number of avian predator species present in the viper habitats and found evidence (pellets, direct observations) of predation on vipers by several raptor species. Second, we found a relatively high proportion (12.5%) of injured *V. graeca* individuals, with more injuries on the posterior than on the anterior body parts. Third, the incidence of predation-related injuries increased with SVL, were more frequent on females than on males and they occurred in shorter SVL for males than for females. Finally, two results suggested that vipers may adjust their diurnal period of activity due to predation because (i) their daily activity was bimodal, probably to avoid the mid-day peak in raptor activity, and (ii) there was only moderate overlap with predicted potential activity because the observed activity of vipers shifted earlier in the morning and later in the afternoon than could be expected based only on thermal conditions. These differences in activity patterns were consistent in two large populations on separate mountain ranges.

We directly observed three avian predator species to consume vipers (*C. cornix*, *C. gallicus*, *F. tinnunculus*). Based on the literature review, this is the first documented case of predation by *C. cornix* on snakes, even though this species is a generalist predator that has been studied mainly in urban environments [63, 64]. In addition, five other reptile-specialist predators were also regularly observed in the viper habitats, and we also obtained evidence on predation on vipers by finding *V. graeca* remains (scales) in faeces of two mammal species (Eurasian Badger *Meles meles* and Red Fox *Vulpes vulpes*).

With regard to the large number of predators, it is not surprising that a relatively large proportion of viper individuals had injuries, i.e., signs of past failed attempts at predation. It is surprising, however, that so many of viper individuals were able to survive predator attacks. Most of these injuries were on the posterior body parts, which likely indicates that the individuals were actively escaping from the predators. The shortage of injuries on anterior body parts indirectly suggests that predation attempts are probably more successful if predators can get a hold of the head or the neck of the viper.

We did not find injuries on juvenile vipers and the incidence of injuries increased with body length, which can can be explained by several, mutually non-exclusive mechanisms. Simply, adult snakes had more time to get injured than juveniles or juveniles may be easier to catch thus they are less likely to survive attacks [65]. The preferred body temperature of juveniles might be lower than that of adults, which can serve as an antipredator adaptation [66]. Also, juveniles may spend less time basking, again, as an antipredator tactic, trading heat for safety [23]. Another alternative explanation is that juvenile snakes are too small to be worth hunting by larger raptors, as was found in a study using plasticine models [67]. Females were also more likely to get injured and they did so at a longer SVL than males. Two explanations for this difference can be that (i) gravid females spend more time sunbathing than males [68, 69] and (ii) gravid females carrying offspring (*V. graeca* is viviparous) can be slower to escape, which may increase their exposure to predators. Again, an alternative explanation is if males, that are usually smaller than females, are more often the victims of successful predation attempts, when predators take the entire individual.

To avoid predators, animals use different strategies, and previous studies have shown that the choice of a thermoregulatory period can be part of a predator avoidance strategy in reptiles [70]. Our results suggest that the daily activity peaks of vipers are shifted towards thermobiologically suboptimal periods to minimise overlap with the activity peak of predators, which can be a predator avoidance strategy. In the summer, *V. graeca* usually bask for approximately 1–2 h after sunrise, which is the best time period to find individuals compared to other times of the day. Thermal updrafts arrive from the valleys 2–3 h after sunrise, which soaring birds of prey exploit to fly up to viper habitats on the mountain. The overlap between the sunbathing period and the appearance of thermal updrafts offers the best chances of preying on vipers for predators because *V. graeca* individuals tend to retreat to their burrows later due to increasing soil and air temperature and/or the appearance of predators. In the late afternoon, when the air cools back, vipers have a second, smaller peak of activity just before sunset, when a smaller number of individuals come out of their burrows for sunbathing and/or feeding than in the morning. While the activity peaks of both vipers and predators can be explained by large-scale patterns in daily temperature, it is important to emphasize that even though periods later in the morning and earlier in the afternoon would similarly be thermobiologically suitable for *V. graeca* for sunbathing, the activity peaks are shifted earlier in the morning and later in the afternoon that could be expected based on temperature changes alone. These patterns indicate that predator avoidance can play a role in the bimodal nature of diurnal activity and the shifting of the activity peaks and that *V. graeca* does not exploit the whole extent of the thermally available activity window, likely due to risks of predation.

Our study offers several novelties in understanding predator–prey relationships involving snakes as prey. This study presents a detailed survey of potential and actual predators of a viper species in open mountain grassland ecosystems based on a large dataset from 14 of 17 known populations of *V. graeca*, covering much of the geographic range of the species. Our most important findings, i.e., the bimodal activity pattern of vipers and the shift in observed activity from the thermobiologically most suitable period to suboptimal periods, are both likely to be influenced by the activity of predators, have not been demonstrated in snakes before.

However, most importantly, our data on injuries may not be complete to assess predation pressure or the full spectrum of predation patterns because we have no information on individuals that perished in successful predation attempts. For example, if most of the predation attempts on juveniles or smaller males were successful, it may lead to the observed overrepresentation of predation-related injuries on females, whereas in reality, females may be better at escaping from predators. Overall predation pressure is probably greatly underestimated by the injury-based method and many of the detected differences can be explained in either of two ways, as elaborated above, because we do not know anything about individuals suffering successful predation attempts. More detailed observation of predators and, if possible, predation events or evidence from predation events such as scales in pellets or faeces are necessary to assess predation pressure and its population-level consequences. Experimental studies using clay or plasticine models would further inform us about the relative importance of avian vs. mammalian predation, the spatial and temporal patterns of predation attempts and so on, which would provide a more accurate assessment of predator activity and predation pressure. Similarly, an experimental study based on the observations or measurements of viper behaviour upon the presentation of a predator decoy would throw more light on whether the activity shift occurs due to behavioural responses triggered by perceived predation risk or to daily temperature changes.

## Conclusions

We detected a large number of avian predators present in the habitats of the endangered Greek meadow viper (*Vipera graeca*) and found evidence (pellets, direct observations) of predation on vipers by three of these species. The relatively high proportion (12.5%) of injured *V. graeca* individuals observed suggests high predation pressure. The frequency of injuries was higher on the mid and posterior body part compared to the head and anterior body parts, likely caused by higher foraging success of predators when they target the head of snakes. Our data suggests that the time spent being active on the surface (thermoregulation and foraging) by the snakes has costs: predation-related injuries increased with body length, were more frequent in females than in males and occurred at shorter SVL for males than for females. The high overlap between viper potential activity and predator activity in contrast to the moderate overlap between observed activity and predator activity suggests that there is a trade-off in the time budget of the studied viper species as individuals adjust their diurnal activity period to predator activity. The vipers show a shift in their daily bimodal activity by being active earlier in the morning and later in the afternoon than could be expected based on the thermal conditions only. This finding suggests that vipers do not fully exploit the thermally optimal time window (thermal niche) available to reach their preferred body temperature in a cold environment.

## Methods

### Study species

The prey species in this study is *Vipera graeca*, which is among the least known endangered snake species in Europe [71]. The 17 known populations of *V. graeca* are found in subalpine meadows above the tree line, between 1,600–2,200 m above sea level in isolated ranges of the Pindos Mountains in southern Albania and central Greece [72]. *V. graeca* is the smallest viper of Europe, the body length of adult individuals averages 35–40 cm, with a maximum of 45 cm, and females are larger than than males. These grassland snakes are dietary specialists on locusts and bush-crickets [73]. Due to climate change and unsustainable land use, approximately 90% of the current habitats are likely to become unsuitable by the 2080s, thus conservation actions need to be implemented urgently to avoid extinction [72].

### Data collection

We collected data in 14 of the 17 known populations of *V. graeca*. Exact locations are not given due to conservation reasons but are available from the corresponding author upon reasonable request. In each population, we intensively searched for snakes during the vipers’ active season between April and September in 2010–2019. We carefully checked the captured individuals for wounds and injuries that potentially originated from attacks of predators and recorded both the sex and the snout-vent length (SVL). After data collection, all individuals were released at the exact location of capture.

To assess the daily activity of vipers, we collected data on the thermoregulation and the thermal environment in two of the largest populations, on Tymfi and Lakmos mountains in Greece in July and August of 2017. We chose these summer months because the species is most active and easiest to capture in this period based on our previous experience. To capture snakes, we searched characteristic *V. graeca* habitats throughout the day as described in Mizsei et al. [71]. We then measured the selected body temperature (T_b_) of captured snakes in 100 × 30 × 30-cm terraria in which we established a thermal gradient ranging from 20 to 40 °C and measured T_b_ hourly by a Testo 826-T4 thermocouple inserted in the snakes’ cloaca. We collected environmental temperature data called operative temperature (T_o_), the temperature that a non-thermoregulating animal could attain based on heat radiation, conduction, and convection. We measured T_o_ by using a physical model made of copper that mimicked the size, shape, and heat absorption of the study species. To measure T_o_, we equipped the models with temperature data loggers (Thermochron iButton DS1921G-F5#) pre-set to record data in 5-min intervals and placed the models in four different micro-environments: in burrows, under rocks, in shade of vegetation, and on soil exposed to sun. The models were placed in the micro-environments closest to the exact location of capture of *V. graeca* individuals.

During snake searches, we also surveyed avian predators in the snake habitats to assemble checklists of avian predators observed in each studied population. During fieldwork to capture viper individuals, we also collected scat samples of mesopredator mammals and pellets from birds of prey, in all of the studied viper populations. To collect pellets of birds, we checked the rocks used as look-out points or resting spots if there were accessible by foot. In addition, we reviewed the literature on the dietary preferences of the observed predator species. To obtain detailed data on the daily activity of predators, we recorded bird activity in both the Tymfi and Lakmos mountains during the peak activity of vipers for 10–10 days in July and August of 2017. To record predator activity, two observers watched for potential predator birds from a mountain peak in each studied location, which enabled us to simultaneously monitor approximately 100 ha area of viper habitat.

### Data analysis

To assess the age and sex distribution of injuries, we used SVL as a proxy for the age of individuals. We fitted negative binomial generalized linear mixed models (glmm) to analyse the effect of SVL and sex on the presence of injuries using the ‘lme4’ package of R [74]. The binary dependent variable was the presence-absence of injuries, while the explanatory variables were the sex and SVL of the individuals. We controlled for potential spatial bias by including population identity as a random factor. We used hazard functions to compare the probability of getting injured for male and female vipers with smoothing spline functions of the ‘gss’ package of R [75].

We estimated activity patterns by probability density functions (PDF) by kernel density estimation or by fitting parametric trigonometric sum distributions of observation time, which was regarded as a circular random variable where the underlying density was expected to be bimodal. We created three sets of PDFs for the Tymfi and Lakmos study sites separately: (1) the daily activity of raptors based on observation times, pooled across the species known to be preying on *V. graeca*; (2) the observed daily activity of *V. graeca* based on observation times; (3) the potential daily activity of *V. graeca* based on thermoregulation measurements. To predict the potential activity of *V. graeca,* we first fitted a function to the frequency of T_b_ values selected in the thermal gradient by viper individuals using the ‘rpearson’ function of the ‘PearsonDS’ package [76], based on the variance, skewness and kurtosis of data, which were estimated by the ‘descdist’ function of the ‘fitdistrplus’ package [77]. We scaled the estimates of the fitted distribution to range between 0–1 and regarded this as activity probability. Second, we calculated the average T_o_ for each 5-min interval, and joined these data with the activity probability values. To obtain time data representing the potential activity of *V. graeca*, we randomly resampled the time of mean T_o_ by regarding activity probability as a probability weight by the ‘sample’ base function of R. We measured the overlap of two activity patterns by calculating the coefficient of overlapping (Δ) using the ‘overlapEst’ function of the ‘overlap’ package [78], which is the area under the curve that is formed by taking the minimum of the two density functions at each time point. Δ can range between 0 (no overlap) and 1 (complete overlap), and is interpreted as the proportion of activity that differs between the two activity patterns by less than 1-Δ in any time period. We calculated the 95 percent confidence intervals for Δ as percentile intervals from 1000 bootstrap samples. All data processing and analyses were implemented in the R 4.0.2 statistical environment [79].

## Data Availability

All data and R codes produced during the preparation of this manuscript are available and deposited at Zenodo repository (10.5281/zenodo.7915826).
